# Optimizing Corn Crop Protection: The First Sampling Plan for Controlling *Dalbulus maidis* (Hemiptera: Cicadellidae)

**DOI:** 10.3390/plants13131779

**Published:** 2024-06-27

**Authors:** Cleovan Barbosa Pinto, Daiane das Graças do Carmo, Juliana Lopes dos Santos, Emílio de Souza Pimentel, Aline da Silva Mota, Ricardo Siqueira da Silva, Marcelo Coutinho Picanço, Renato Almeida Sarmento

**Affiliations:** 1 Graduate Program in Biotechnology and Biodiversity, Rede Bionorte, Federal University of Tocantins, Palmas 77650-000, TO, Brazil; cleovannat@hotmail.com (C.B.P.); julianalopes@uft.edu.br (J.L.d.S.); 2 Department of Plant Science, Universidade Federal de Viçosa, Viçosa 36570-900, MG, Brazil; daiane.carmo18@gmail.com; 3 Department of Entomology, Universidade Federal de Viçosa, Viçosa 36570-900, MG, Brazil; emilio.pimentel@ufv.br; 4 Graduate Program in Plant Production, National Institute of Science and Technology on Terrestrial Ecotoxicology, Federal University of Tocantins, Gurupi 77402-970, TO, Brazil; silva.mota@mail.uft.edu.br; 5 Department of Agronomy, Universidade Federal dos Vales do Jequitinhonha e Mucuri, Diamantina 39100-000, MG, Brazil; ricardo.siqueira@ufvjm.edu.br

**Keywords:** *Zea mays*, corn leafhopper, sampling time, sampling cost, negative binomial

## Abstract

Corn (*Zea mays*) is the most widely planted crop in the world. *Dalbulus maidis* (Hemiptera: Cicadellidae) is currently a primary corn pest. The starting point for the development of pest control decision-making systems is the determination of a conventional sampling plan. Therefore, this study aimed to determine a practical conventional sampling plan for *D. maidis* in corn crops. Insect density was evaluated in 28 commercial fields. Subsequently, *D. maidis* densities were sampled from fields ranging from 1 to 100 ha. Insect density conformed to a negative binomial distribution in 89.29% of the fields. The insect densities determined using the sampling plan had a low error rate (up to 15%). Sampling time and costs ranged from 2.06 to 39.45 min/ha and 0.09 to 1.81 USD/ha for fields of 1–100 ha, respectively. These results provide the first precise and representative conventional sampling plan for scouting *D. maidis* adults grown in corn fields. Therefore, the conventional sampling plan for *D. maidis* determined in this study is practical and can be incorporated into integrated pest management programs for corn crops owing to its representativeness, precision, speed, and low cost.

## 1. Introduction

Corn (*Zea mays* L. (Cyperales: Poaceae)) is the most produced grain in the world, serving as a fundamental food source for both human and animal nutrition, and is utilized in biofuel production [1,2]. Corn is currently cultivated in 164 countries across all continents. In 2022, a total of 203.47 million tons of corn were produced, covering an area of 1.16 billion ha [1]. In Brazil, corn-planted area covered 22.5 million ha in 2022–2023 and corn production was 125.5 million metric tons [1].

The corn leafhopper *Dalbulus maidis* (DeLong and Wolcott) (Hemiptera: Cicadellidae) is currently the primary pest of corn crops in regions where this insect is present [3,4]. Reported losses of up to 70% in corn crop productivity have been attributed to direct and indirect damage caused by *D. maidis* [3,4]. *D. maidis* is found in South America, Central America, and North America [5]. Adults of *D. maidis* are approximately 4 mm in length and straw-yellow in color, with two circular black spots on top of their heads, and females lay eggs endophytically on the leaves [4].

This pest can cause both direct and indirect damage to corn plants. Direct damage occurs as nymphs and adults feed on the sap and inject toxins into plants. Indirect damage is attributed to the transmission of pathogen-based diseases, such as *Spiroplasma kunkelii*-caused maize stunt spiroplasma (CSS), maize bushy stunt phytoplasma (MBSP), and maize rayado fino virus (MRFV) [3,6,7]. The critical period for pest control is during the vegetative stage of plants [3,8]. However, no economic threshold has been established for this pest. *D. maidis* is a highly efficient pathogenic vector. Therefore, control programs for this pest are not based on any economic threshold but on the mere presence of this insect in crops [9]. This low tolerance can lead producers to excessively apply insecticides per crop cycle, which can have negative effects on natural enemies [9,10].

In the management of *D. maidis* in corn crops, resistance of plants, cultural methods, chemical control, and biological control can be employed. When dealing with insect vectors and their associated problems, it is essential to apply efficient and fast-acting methods to reduce pathogen transmission to plants [3,11,12,13]. The insecticides most commonly used to control *D. maidis* are neonicotinoids (thiamethoxam, acetamiprid, zimidacloprid, and clothianidin), pyrethroids (bifenthrin, beta-cyfluthrin, lambda-cyhalothrin, and esfenvalerate), and organophosphates (profenofos, acephate, and fenitrothion) [14], and biological control with *Beauveria bassiana* and *Metarhizium anisopliae* is also widely used [15]. The main disadvantage in the chemical control of *D. maidis* does not appear to be the effectiveness of the available active ingredients but rather their speed of action and the necessity to spray multiple times when the timing of the first spray is not ideal [9].

The adoption of a decision-making system is necessary to enable farmers to effectively control pests. The starting point for creating pest control decision-making systems is the determination of conventional sampling plans. This plan comprises the sampling unit and technique, the number of samples, sampling time, and cost [16,17,18]. In this context, Pinto et al. [19] determined an ideal sample and technique for sampling *D. maidis* in corn crops, with whorl leaves as the most suitable sampling unit for evaluating these pest populations and direct counting as the best technique. This is the only current sampling recommendation for this species. Despite the significance of *D. maidis* in cornfields, no conventional sampling plan has been established for this pest.

Sampling plans must be accurate, representative, and practical. The components of sampling plans (the sample unit, sample size, and sampling technique) are considered accurate when the densities are determined with a relative variance of less than 25% [12,17,20]. Furthermore, these components are deemed representative when the determined relative densities have positive and significant correlations with absolute pest densities in the evaluated fields [12,21]. The samples and techniques selected by Pinto et al. [19] for *D. maidis* sampling in cornfields exhibited these characteristics.

Sampling plans are considered practical if a simple and uniform methodology is adopted throughout the plant development period. Moreover, they should be quick to execute and incur low costs. Owing to these characteristics, farmers are more likely to adopt these sampling plans [22,23]. According to Gusmão et al. [24], a sampling plan is deemed practical when it can be executed in one morning or afternoon. This allows for quick control decisions before a pest causes economic damage. Thus, the objective of this study was to determine a practical conventional sampling plan for *D. maidis* in corn crops that could be incorporated into integrated pest management programs in corn fields. The hypothesis of this study was that a practical and precise conventional sampling plan for *Dalbulus maidis* in corn crops could be developed and incorporated into integrated pest management programs.

## 2. Results

### 2.1. Frequency Distribution of Dalbulus maidis Densities

In 25 of the 28 corn crops, the *D. maidis* densities conformed to a negative binomial frequency distribution; in these crops, the chi-squared values were not significant (*p* > 0.05; Table 1). The *D. maidis* density followed a Poisson distribution in 10 of the 28 corn fields. Only in 4 of these 28 corn fields did the *D. maidis* density fit a positive binomial distribution, with non-significant χ^2^ values (*p* < 0.05; Table 1). Therefore, the number of samples for the *D. maidis* sampling plan was calculated using the formula for negative binomial frequency distribution, as most crops conformed to this distribution.

### 2.2. Number of Samples for the Sampling Plan

The regression curve of the aggregation parameter (K_common_) of the 28 corn crops as a function of the K parameter of each crop showed a significant slope (*p* < 0.05) and non-significant intercept (*p* > 0.05; Table 2). Therefore, there was a common aggregation parameter (K_common_ = 0.7836) between *D. maidis* densities in all corn crops.

**Figure 1:**
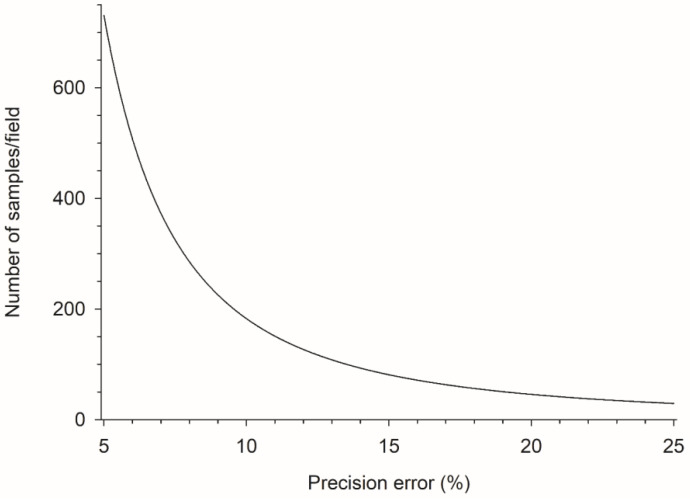
Number of samples to evaluate *Dalbulus maidis* density as a function of sampling error in corn crops.

The number of samples in the sampling plan as a function of error stabilized when an error of 15% was reached. Therefore, an error of 15% was used to calculate the number of samples required for the sampling plan. This value allowed for the generation of a viable sampling plan for crops of different phenological stages in two different biomes: the Atlantic Forest and Cerrado. Using a maximum error of 15% and $\bar{x}\;$= 1.84 (with variation from 0.01 to 12.65) *D. maidis*/plant, 81 samples were required to compose an efficient sampling plan (Figure 1).

### 2.3. Time and Costs Required for Sampling

The distance covered and walking time during *D. maidis* sampling varied according to the size of the corn fields. The distances covered to sample crops of different sizes and biomes ranged from 1509.55 to 15095.45 m. The time spent walking between the sampling points ranged from 18.50 to 185.52 min. The time required to evaluate 81 samples was 20.95 min (sample time to evaluate whole leaves without considering walking or noting time). Thus, the total time required for sampling *D. maidis* ranged from 39.45 to 206.47 min (sample assessment + walking time; Table 3). Total costs for *D. maidis* sampling ranged from USD 1.81 to USD 9.46 (Table 3).

## 3. Discussion

This study proposes the first precise and representative conventional sampling plan to scout corn leafhopper *D. maidis* adults in corn fields.

The densities of *D. maidis* conformed to a negative binomial distribution in most corn crops (89.28%). This type of distribution occurs when the variance exceeds the mean density [18,25,26]. In our study, this occurred due to the high frequency of samples with extreme densities, which caused the variances to be greater than the means. Sample distribution does not provide information about the spatial distribution of insects because it does not consider the location of the sampled plants [27,28].

Thus, the number of samples required to establish a conventional sampling plan for adults and nymphs of *D. maidis* was calculated using the formula reported by Young and Young [29] for a negative binomial distribution. The densities of *D. maidis* shared a common aggregation parameter among different corn crops. This indicates that the sampling plan proposed in this study is applicable to multiple commercial corn fields [21,25,30]. In addition, a common aggregation parameter was generated for crops at different phenological stages, sizes, and biomes with different infestation levels. This makes the sampling plan for *D. maidis* practical and more likely to be adopted by corn producers.

Sampling plans for insect pests should be feasibly implementable [17,25]. A sampling plan is considered viable when it is accurate, simple, quick, and inexpensive [25,31]. The maximum allowed error for a sampling plan is 25%, and the lower the error value, the higher the accuracy of the pest density measurements [32]. A plan is considered fast when data collection in the field, data processing, and decision-making are completed within one day [18,21]. This is important for the rapid implementation of control measures. Thus, the application of this sampling plan is highly feasible, as it allows decision-making within less than a day (maximum, 206.47 min) and costs less than USD 10.00 for fields up to 100 ha in size. Virla et al. [13] observed that a density of 10 *D. maidis* individuals is sufficient to cause damage to 10-day-old seedlings, even under irrigation; however, under drought conditions, damage can be significantly greater, causing plant mortality. *D. maidis* is responsible for 10–100% of plants with symptoms of maize-stunting pathogens, which can lead to complete production losses [33]. Therefore, the damage caused by this insect justifies the adoption of this sampling plan to avoid yield losses.

The walking time between samples and plot size strongly influences the sampling time. In most cases, up to 60% of the time is spent walking, reaching 89.85% in 100 ha plots [34]. However, the scouting time for evaluating all 81 samples was short (20.95 min). *Dalbulus maidis* exhibits edge effects, with the highest infestations occurring at the center pivot boundaries of corn fields [35]. To date, no studies have been conducted on whether the same occurs in regular fields without irrigation. Therefore, we established a limit of 50 m from the edge to prevent this effect from influencing the sampling plan.

The next step in this study was to establish an economic threshold and a sequential sampling plan for this important corn pest. However, no level of economic damage caused by *D. maidis* has been established. Its control is based solely on the presence of pests and the application of insecticides throughout the field [16].

Additionally, the adoption of a sampling plan in pest control decision-making systems can reduce the use of insecticides in cornfields by providing more information for appropriate pest-management decisions [17,34]. This reduces environmental contamination and the exposure of workers. Moreover, it contributes to the preservation of non-target organisms such as natural enemies and pollinators. Correct use of insecticides also reduces the selective pressure that can lead to *D. maidis* populations becoming resistant to these pesticides [17,34,36].

## 4. Materials and Methods

### 4.1. Experimental Conditions

The study was conducted in two stages: during the first stage, the number of samples for the sampling plan was determined, and during the second stage, the sampling times and costs were determined. The densities of *D. maidis* were assessed during the years 2020–2021 and 2021–2022 in commercial corn fields (varieties P3858PWU and P3707VYH Pioneer^®^ Seeds) conducted in the biomes of the Atlantic Forest (20°45′44″ S, 42°52′12″ W) and Cerrado (11°44′44″ S, 49°02′58″ W) in Brazil. *D. maidis* densities were evaluated weekly until the end of the vegetative stage (VT) when the corn developed a tassel.

Crop fertilization was performed based on soil chemical analysis. Irrigation, insecticides, and fungicides were not used, and weed control was achieved using herbicides. Normal practice is to apply at least three sprays of insecticides and fungicides [37]. However, to avoid affecting the *D. maidis* density, this study was conducted in fields where no insecticides were sprayed.

### 4.2. Frequency Distribution of Dalbulus maidis Densities

**Figure 2:**
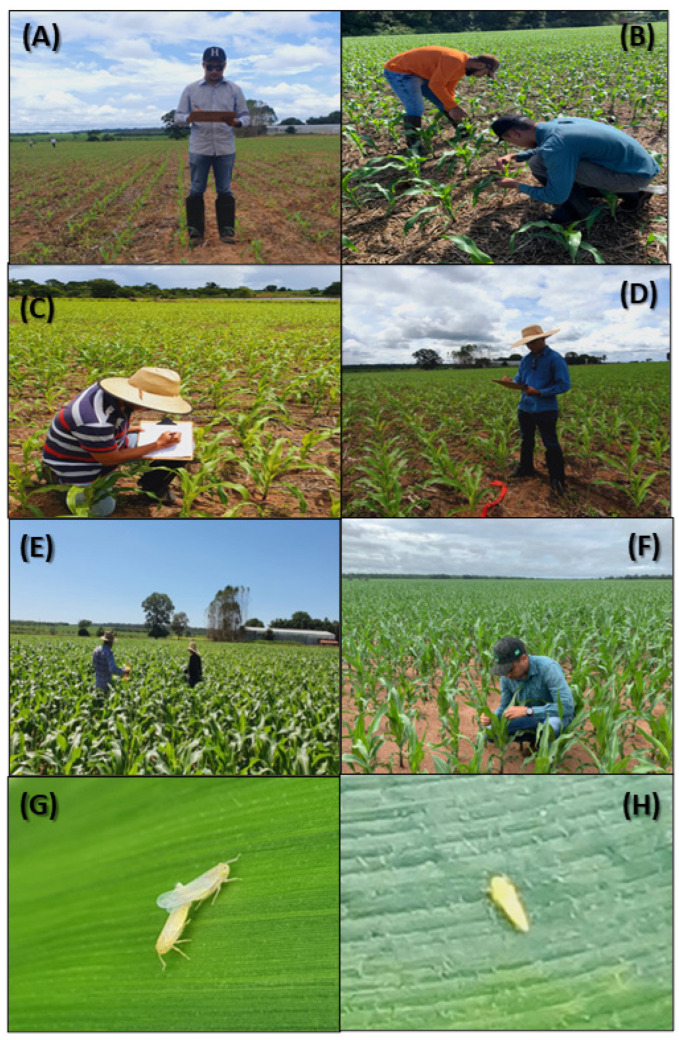
(**A**) V4, (**B**) V6, (**C**) V8, and (**D**) V10 plant stages. (**E**) Walking in corn fields and (**F**) sampling of *Dalbulus maidis.* (**G**) *D. maidis* adult and (**H**) nymph.

This phase of the study was conducted in 28 commercial corn fields, with each cultivation field containing 197–250 evaluated plants. Plants were evenly distributed to avoid directional biases [12,25,38]. The distance from the edge of the fields was consistent between the sampling points (50 m from the edge). The distance between samples was determined based on the size of the field. The size of the sampled fields ranged from 3 to 30 ha. Fields with plants at different phenological stages (V4, V6, V8, and V10) were evaluated to ensure that the sampling plan determined in this study was applicable for the entire corn cultivation period (Figure 2). In each field, plants distributed throughout the entire area were assessed, and all leaves on each plant were examined. The age of the plants (in days), phenological stage, and density of *D. maidis* (adults and nymphs) were recorded. It takes approximately 100–120 days from planting to the harvesting of corn, and the attack by *D. maidis* begins when the crop emerges [39]. The critical period lies between emergence and the V8 stage [3]. *D. maidis* densities were sampled from whorl leaves using the direct counting technique, which was chosen as the ideal method by Pinto et al. [19]. Densities (mean ± standard error) of *D. maidis* adults were calculated for each field.

The observed and expected frequencies were calculated using negative binomial, Poisson, and positive binomial distributions [17,21,39]. The observed and expected frequencies were compared using the chi-square test (χ^2^). The data on *D. maidis* densities in a crop were deemed to fit a frequency distribution when the χ^2^ values of the observed and expected frequencies were not significant (*p* > 0.05). The frequency distribution that best fit the pest densities of most crops was selected [21,40,41].

### 4.3. Number of Samples for the Sampling Plan

After the pest densities conformed to a negative binomial distribution, the values of the common aggregation parameter (K_common_) for each crop were calculated using Equation (1) [29]:

(1) $$k={\bar{x}}^{2}/\left(S^{2}-\bar{x}\right)$$

where *k* is the parameter of the negative binomial distribution, ${\bar{x}}^{2}$ is the mean, and $S^{2}$ is the variance in the density of *D. maidis*.

The *k* value and *D. maidis* average density for each crop were subjected to simple linear regression analysis using a procedure described by Bliss and Owens [30]. In this analysis, fields had K_common_ when the equation presented a significant slope and a non-significant intercept according to the F-test at *p* < 0.05 [30].

After calculating K_common_, the number of samples required from the *D. maidis* sampling plan for corn crops was estimated. The number of samples was calculated using Equation (2) [12,18,29]:

(2) $$NA=\frac{1}{C^{2}}+\left(\frac{1}{\bar{x}}+\frac{1}{kc}\right),$$

where *NA* is the number of sampling units, *C* is the admitted error, $\bar{x}$ is the population mean, and *kc* is the common aggregation parameter.

These calculations were performed using sampling errors of 5%, 10%, 15%, 20%, and 25% because this is the error range (5–25%) used to calculate estimates of average insect densities in decision-making systems for integrated pest management programs [25,32]. A graph was created for the number of samples as a function of sampling error. The error used in the final calculation of the number of samples in the sampling plan was the error from which the number of samples was stabilized [16,21,24].

### 4.4. Determination of Sampling Plan Time and Costs

Subsequently, *D. maidis* densities were sampled in eight commercial corn fields with areas of 1, 3, 5, 10, 20, 30, 50, and 100 ha and with plants at different phenological stages (V4, V6, V8, and V10). Sampling was conducted using a previously determined number of samples. For each field, we recorded the distance covered, total sampling time, and time spent walking between samples. The distance covered and walking time during *D. maidis* sampling varied according to the size of the corn fields. The distance between the samples was determined based on the size of the field; therefore, 81 samples were equidistantly distributed, and the entire field was sampled. The cost of sampling *D. maidis* for each field was also calculated. These calculations considered the costs of materials necessary for sampling (rubber, pencil, paper, clipboard, and white plastic tray), as well as the cost of salaries and social security contributions for rural workers [18,25,42] (This cost was estimated based on the salary in Brazil, with a salary/h of 2.09 ISD/h with the dollar exchange rate of BRL 5.15).

## 5. Conclusions

Therefore, the first conventional scientific sampling plan for *D. maidis* proposed in this study is practical and can be incorporated into integrated pest management programs for corn crops owing to its representativeness, precision, speed, and low cost. This study can also serve as a basis for the development of economic thresholds and sequential sampling plans. This is our contribution to more sustainable agriculture without the unnecessary spraying of insecticides to control this pest.

## Figures and Tables

**Table 1:** Probability distribution of *Dalbulus maidis* density per plant in 28 corn fields in two biomes and at various plant stages for two years.

Fields	N	Density	Negative Binomial		Poisson		Positive Binomial	
			χ^2^	df	χ^2^	df	χ^2^	df
1	197	0.04 ± 0.01	1.76 × 10^−4 ns^	1	8.92 × 10^−3 ns^	1	3.38 ^ns^	1
2	197	0.05 ± 0.02	2.25 × 10^−4 ns^	1	1.90 × 10^−2 ns^	1	4.30 *	1
3	197	0.02 ± 0.01	1.02 × 10^−4 ns^	1	1.66 × 10^−3 ns^	1	1.96 ^ns^	1
4	221	0.04 ± 0.01	1.60 × 10^−4 ns^	1	1.06 × 10^−2 ns^	1	3.86 *	1
5	221	0.05 ± 0.02	3.16 ^ns^	1	2.31 ^ns^	1	1.41 × 10^1^ *	1
6	221	0.13 ± 0.03	1.93 ^ns^	2	5.38 × 10^2^ *	3	9.32 × 10^1^ *	3
7	233	0.61 ± 0.08	5.69 ^ns^	4	6.09 × 10^2^ *	5	3.07 × 10^3^ *	5
8	233	0.34 ± 0.07	2.15 ^ns^	3	2.10 × 10^5^ *	4	7.84 × 10^2^ *	4
9	233	0.01 ± 0.01	3.68 × 10^−5 ns^	1	1.48 × 10^−4 ns^	1	0.99 ^ns^	1
10	233	0.01 ± 0.01	3.68 × 10^−5 ns^	1	1.48 × 10^−5 ns^	1	0.99 ^ns^	1
11	221	0.20 ± 0.05	2.32 ^ns^	5	5.67 × 10^4^ *	6	3.75 × 10^2^ *	6
12	221	0.11 ± 0.03	2.07 ^ns^	1	1.04 × 10^2^ *	2	5.33 × 10^1^ *	2
13	221	0.01 ± 0.06	4.09 × 10^−5 ns^	1	1.64 × 10^−4 ns^	1	5.33 × 10^1^ *	1
14	250	0.04 ± 0.01	1.56 × 10^−4 ns^	1	1.62 × 10^−2 ns^	1	4.81 *	1
15	250	0.26 ± 0.05	2.12 ^ns^	3	5.20 × 10^2^ *	4	6.68 × 10^3^ *	4
16	250	1.46 ± 0.15	9.10 ^ns^	9	4.84 × 10^4^ *	10	4.67 × 10^5^ *	10
17	250	0.36 ± 0.04	2.32 ^ns^	1	1.40 ^ns^	2	4.80 × 10^2^ *	2
18	250	11.42 ± 0.57	1.04 × 10^1 ns^	23	5.38 × 10^4^ *	24	3.56 × 10^80^ *	24
19	250	4.90 ± 0.32	1.45 × 10^1 ns^	12	1.91 × 10^3^ *	13	2.64 × 10^31^ *	13
20	250	12.65 ± 0.49	3.97 × 10^1^*	23	3.41 × 10^4^ *	24	9.10 × 10^66^ *	24
21	250	0.39 ± 0.05	2.40 ^ns^	2	3.10 × 10^1^ *	3	4.24 × 10^2^ *	3
22	250	4.62 ± 0.28	1.86 × 10^1 ns^	16	2.38 × 10^4^ *	17	3.18 × 10^30^ *	17
23	250	1.89 ± 0.18	1.66 × 10^12^ *	10	8.72 × 10^4^ *	11	8.20 × 10^6^ *	11
24	250	0.07 ± 0.02	1.44 × 10^−2 ns^	1	2.96 × 10^−1^ *	1	2.42 × 10^1^ *	1
25	250	0.26 ± 0.05	3.99 × 10^−1 ns^	2	1.29 × 10^2^ *	3	3.58 × 10^2^ *	3
26	250	3.95 ± 0.31	2.22 × 10^2 ns^	15	9.59 × 10^5^ *	16	9.04 × 10^23^ *	16
27	250	1.62 ± 0.17	7.38^4 ns^	11	8.77 × 10^5^ *	12	1.18 × 10^9^ *	12
28	250	5.36 ± 0.35	7.41 × 10^15^ *	15	2.43 × 10^6^ *	16	2.89 × 10^26^ *	16

^ns^ Non-significant. * Significant at 5% probability using the F-test.

**Table 2:** Analysis of variance of *Dalbulus maidis* densities in 28 corn crops to assess the existence of a common aggregation parameter (K_common_) using the negative binomial probability distribution.

Factors	Degrees of Freedom	F
Slope 1/K_common_	1	22.46 *
Intercept	1	0.01 ^ns^
Residual	25	

^ns^ Non-significant. * Significant at 5% probability using the F-test.

**Table 3:** Time and costs of the *Dalbulus maidis* sampling plan in corn crops of different sizes.

Field Size (ha)	Distance Walking (m)	Walk between Samples		Sample Assessment		Sampling Time		Cost/Sampling	
		Time (min)	%	Time (min)	%	Min/Field	Min/ha	USD/Field	USD/ha
1	1509.55	18.50	46.89	20.95	53.11	39.45	39.45	1.81	1.81
3	2614.61	32.39	60.72	20.95	39.28	53.34	17.78	2.44	0.81
5	3375.45	41.63	66.52	20.95	33.48	62.58	12.52	2.87	0.57
10	4773.60	58.98	73.79	20.95	26.21	79.93	7.99	3.66	0.37
20	6750.89	83.19	79.88	20.95	20.12	104.14	5.21	4.77	0.24
30	8268.12	101.80	82.93	20.95	17.07	122.75	4.09	5.62	0.19
50	10,674.10	130.92	86.21	20.95	13.79	151.87	3.04	6.96	0.14
100	15,095.45	185.52	89.85	20.95	10.15	206.47	2.06	9.46	0.09

## Data Availability

Raw data supporting the conclusions of this study will be made available by the authors upon request.
